# Supplementary figures and images for: A preliminary study of resting brain metabolism in treatment-resistant depression before and after treatment with olanzapine-fluoxetine combination
Source: PLoS One. 2020 Jan 13;15(1):e0226486. doi: 10.1371/journal.pone.0226486 (PMC6957341; doi:10.1371/journal.pone.0226486)

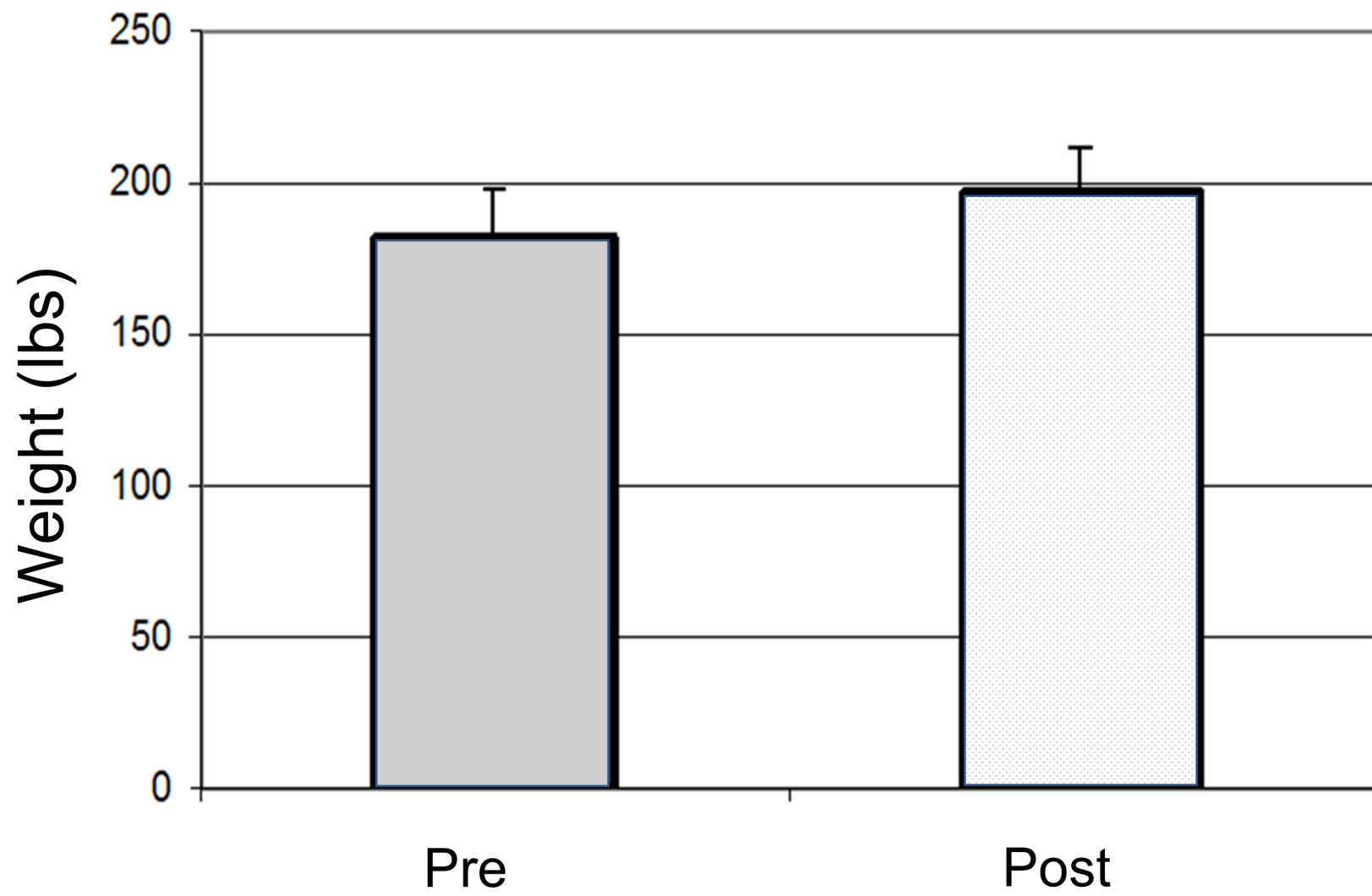

Supplement: S1 Fig — (PDF) [file pone.0226486.s001.pdf]

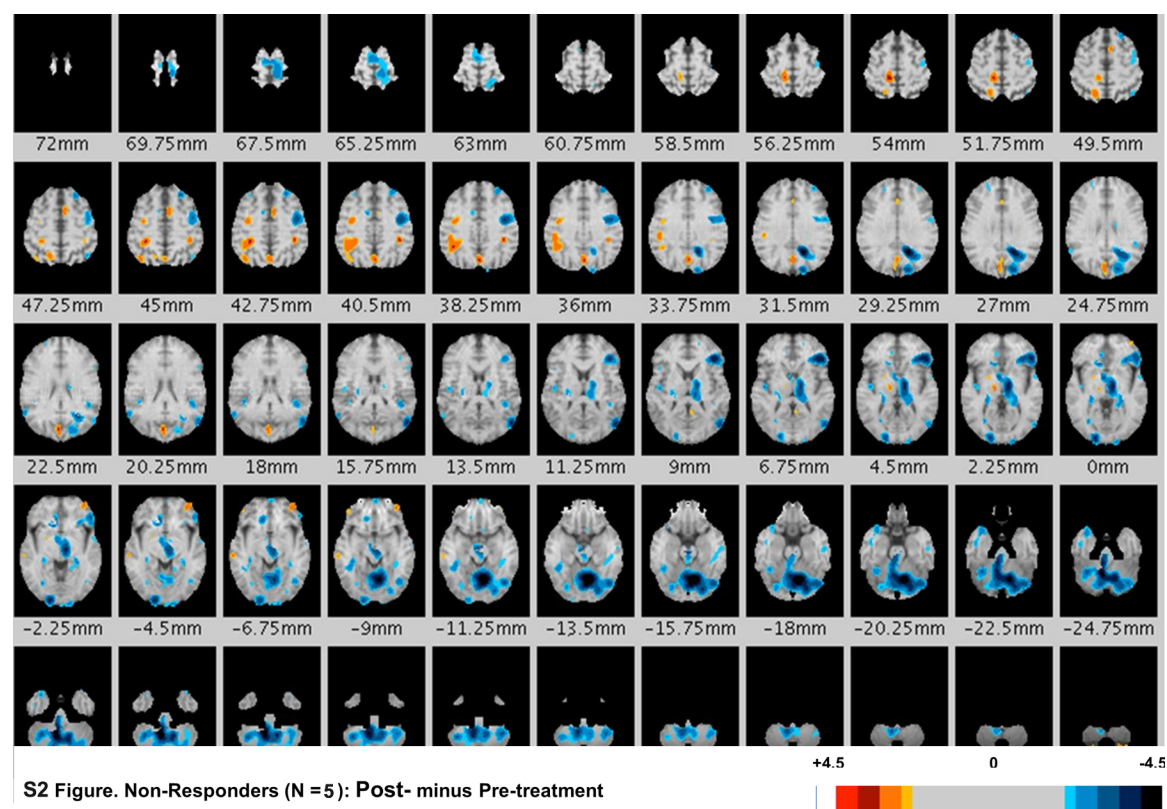

Supplement: S2 Fig — Stereotactically normalized. Image left is right side of brain. AC-PC plane 0 mm. Color scale shows Z-scores with threshold Z = ±3.3. (PDF) [file pone.0226486.s002.pdf]

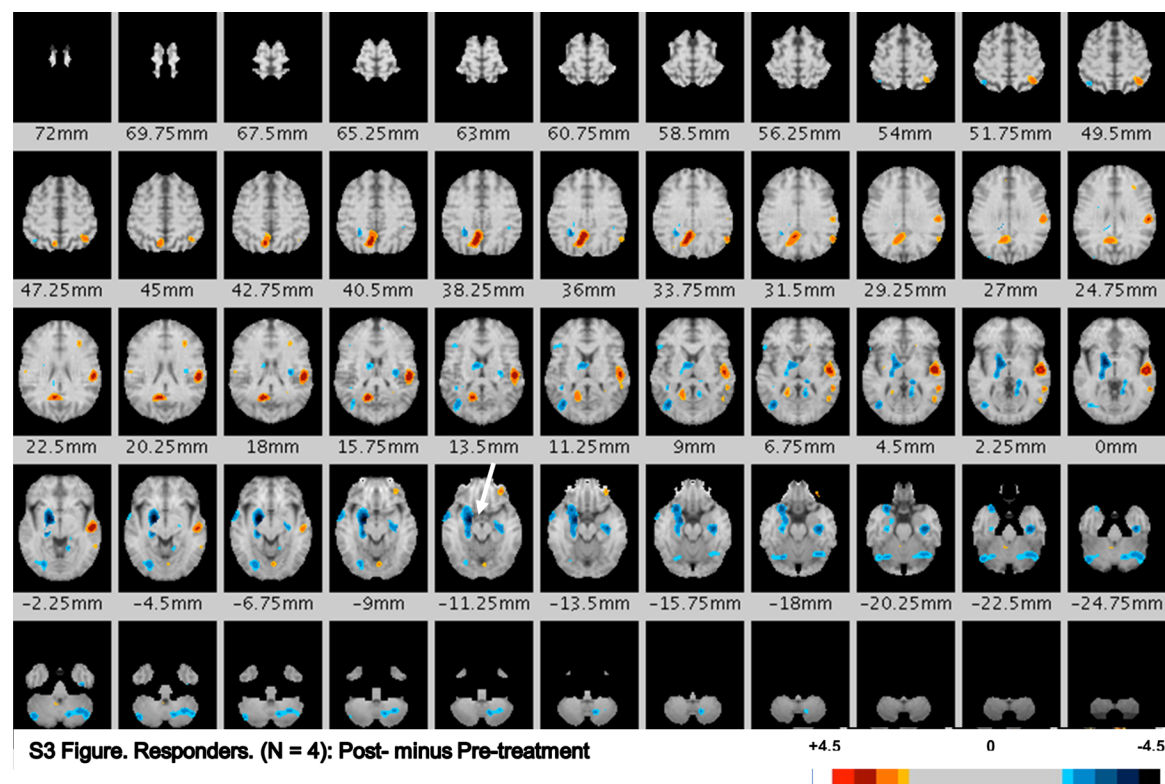

Supplement: S3 Fig — Stereotactically normalized. Image left is right side of brain. AC-PC plane 0 mm. Color scale shows Z-scores with threshold at Z = ±3.3. (PDF) [file pone.0226486.s003.pdf]

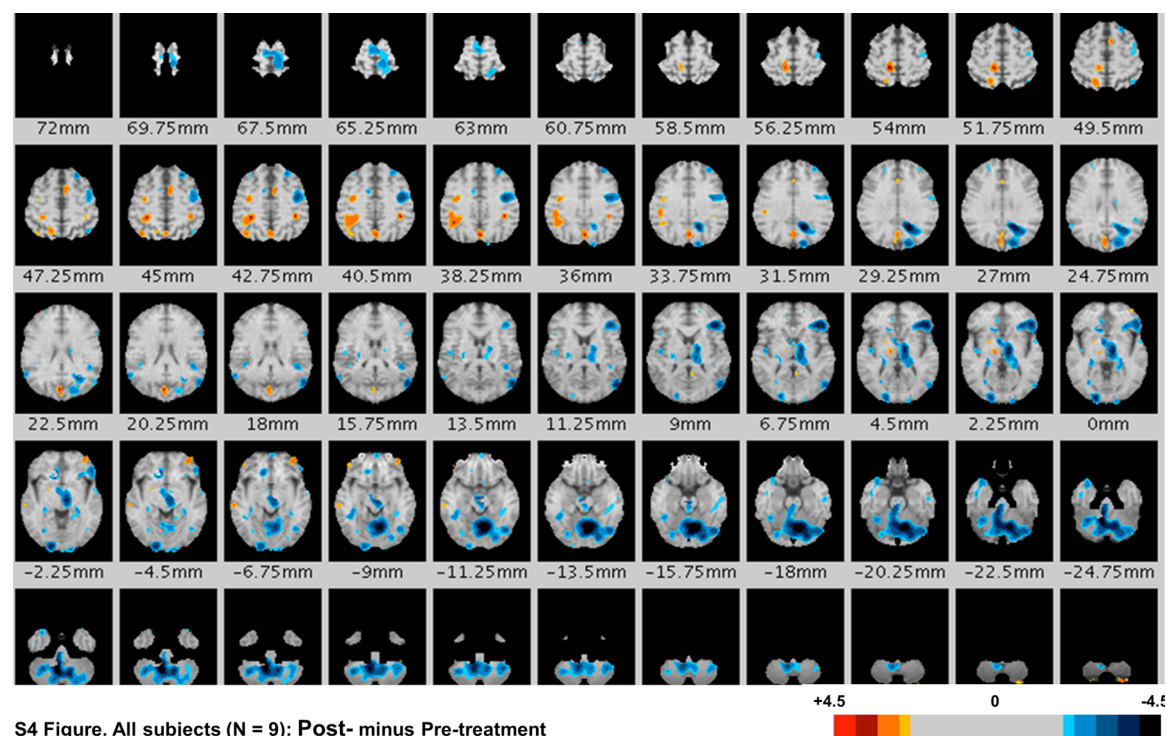

S4 Figure. All subjects (N = 9): Post- minus Pre-treatment

Supplement: S4 Fig — Stereotactically normalized. Image left is right side of brain. AC-PC plane 0 mm. Color scale shows Z-scores with threshold at Z = ±3.3. (PDF) [file pone.0226486.s004.pdf]

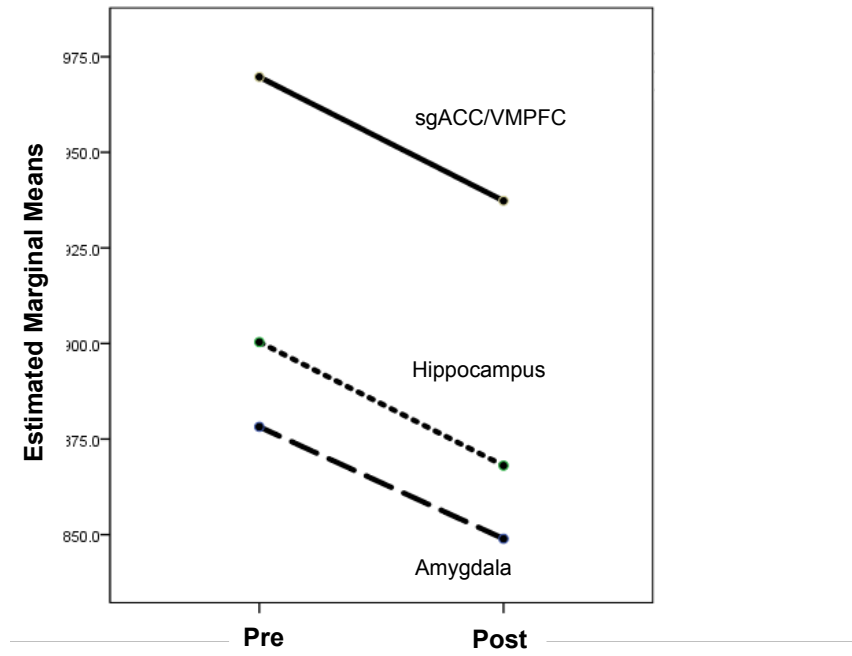

Supplement: S6 Fig — (PDF) [file pone.0226486.s006.pdf]
