# Supplementary material for: A preliminary study of resting brain metabolism in treatment-resistant depression before and after treatment with olanzapine-fluoxetine combination
Source: PLoS One. 2020 Jan 13;15(1):e0226486. doi: 10.1371/journal.pone.0226486 (PMC6957341; doi:10.1371/journal.pone.0226486)
Supplement: S1 Table — (PDF) [file pone.0226486.s007.pdf]

S1 Table. Pre- vs. Post- Weights, depression scores, and anxiety scores.

Scan # pL	Age (years)	Weight pre lb	Weight post lb	MADRS pre	MADRS post	HAMA pre	HAMA post
0020/0021	47	100	132	33	31	25	16
0009/0011	44	279	288	29	24	25	19
0026/0027	52	163	176	37	29	15	8
0028/0029	36	217	235	27	20	9	7
0030/0031	62	185	188	29	12	16	16
0059/0069	54	198	205	24	4	17	11
0071/0072	61	187	210	31	20	12	6
0079/0087	27	162	173	38	19	29	15
0089/0095	41	142	164	28	10	14	10

lb, pounds; MADRS, Madras Asberg Depression Rating Scale; HAMA, Hamilton Anxiety Scale.
