# Supplementary material for: A preliminary study of resting brain metabolism in treatment-resistant depression before and after treatment with olanzapine-fluoxetine combination
Source: PLoS One. 2020 Jan 13;15(1):e0226486. doi: 10.1371/journal.pone.0226486 (PMC6957341; doi:10.1371/journal.pone.0226486)
Supplement: S2 Table — Green cells below diagonal are for Pre-treatment; blue cells above diagonal are for Post-treatment. R, right; L, left; Hippo, hippocampus; sgACC, subgenual anterior cingulate/VMPFC. † P = .007 (PDF) [file pone.0226486.s008.pdf]

S2 Table. Correlation matrix for metabolism in ROIs.

	R amygdala	L amygdala	R Hippo	L Hippo	R sgACC	L sgACC
R amygdala	1	0.611	0.604	0.583	0.074	-0.008
L amygdala	0.011	1	0.241	0.232	0.052	-0.040
R Hippo	0.595	0.173	1	0.449	0.244	-0.031
L Hippo	0.210	-0.033	-0.115	1	0.125	-0.296
R sgACC	0.360	-0.341	0.425	0.004	1	0.821†
L sgACC	0.527	0.502	0.447	0.069	0.283	1

Green cells below diagonal are for Pre-treatment; blue cells above diagonal are for Post-treatment. R, right; L, left; Hippo, hippocampus; sgACC, subgenual anterior cingulate/VMPFC.

† $p < 0.007$
