# Supplementary material for: A preliminary study of resting brain metabolism in treatment-resistant depression before and after treatment with olanzapine-fluoxetine combination
Source: PLoS One. 2020 Jan 13;15(1):e0226486. doi: 10.1371/journal.pone.0226486 (PMC6957341; doi:10.1371/journal.pone.0226486)
Supplement: S1 Data — (PDF) [file pone.0226486.s009.pdf]

Pt	Age yrs.	Sex	PreWeight lb	PostWeight lb	PreMADRS	PostMADRS	PreHamA	PostHamA
LP (pL0009, 0011)	44	M	279	288	29	24	25	16
SB (pL0020, 0021)	47	F	100	132	33	31	25	19
SA (pL0026, 0027)	52	M	163	176	37	29	15	8
JT (pL0028, 0029)	36	M	217	235	27	20	9	7
JW (pL0071, pL0072)	61	F	187	210	31	20	16	16
RO (pL0030, pL0031)	53	M	185	188	29	12	17	11
NH (pL0059, pL0069)	60	M	198	205	24	4	12	6
JS (pL0079, pL0087)	27	M	162	173	38	19	29	15
KS (pL0089, pL0095)	41	M	142	164	28	10	14	10
