# Supplementary material for: A preliminary study of resting brain metabolism in treatment-resistant depression before and after treatment with olanzapine-fluoxetine combination
Source: PLoS One. 2020 Jan 13;15(1):e0226486. doi: 10.1371/journal.pone.0226486 (PMC6957341; doi:10.1371/journal.pone.0226486)
Supplement: S2 Data — (PDF) [file pone.0226486.s010.pdf]

	23,-4,-16	-23-4,-16	27,-23,-9	-27,-23,-9	1,10,-5 to 12,25,-16	-1,10,-5 to -12,25,-16	Coordinate
**study #**	**amygdala, right**	**amydala, left**	**hippocampus, right**	**hippocampus, left**	**subgenual, right**	**subgenual, left**	**ROI**
**pre**							
pL0009	824	872	869	911	886	870	
pL0020	893	898	869	861	882	1005	
pL0026	829	851	935	859	934	956	
pL0028	900	911	934	919	963	1053	
pL0030	978	834	902	913	971	966	
pL0059	947	888	979	895	993	1028	
pL0071	932	848	942	918	1005	995	
pL0079	804	850	806	907	977	962	
pL0089	889	858	934	855	1055	955	
**post**							
pL0011	899	865	850	852	878	916	
pL0021	887	951	903	809	909	958	
pL0027	850	807	871	869	948	951	
pL0029	845	827	919	884	806	794	
pL0031	863	854	823	888	895	931	
pL0069	839	792	888	820	970	1020	
pL0072	912	815	970	893	997	978	
pL0087	750	753	808	779	930	961	
pL0095	862	909	901	897	1052	978	
